# A Simple Model for Assessment of Anti-Toxin Antibodies

**DOI:** 10.1155/2013/230906

**Published:** 2013-06-05

**Authors:** Alex Skvortsov, Peter Gray

**Affiliations:** HPP Division, Defence Science and Technology Organisation, 506 Lorimer Street, Fishermans Bend, VIC 3207, Australia

## Abstract

The toxins associated with infectious diseases are potential targets for inhibitors which have the potential for prophylactic or therapeutic use. Many antibodies have been generated for this purpose, and the objective of this study was to develop a simple mathematical model that may be used to evaluate the potential protective effect of antibodies. This model was used to evaluate the contributions of antibody affinity and concentration to reducing antibody-receptor complex formation and internalization. The model also enables prediction of the antibody kinetic constants and concentration required to provide a specified degree of protection. We hope that this model, once validated experimentally, will be a useful tool for in vitro selection of potentially protective antibodies for progression to in vivo evaluation.

## 1. Introduction

Passive immunization using antibodies has been used successfully for treatment and prophylaxis of infectious disease in humans, and there is increasing interest in the use of antibodies for treatment of infectious diseases that may be used as terrorist weapons, but for which the risk is not sufficiently high to justify preventive vaccination of a large civilian population (see [1–4] and references therein). Toxins are an important potential target for designing therapies against these threats and a broad range of approaches have been taken to develop inhibitors that may be of prophylactic or therapeutic use [1, 5].

Antibody engineering techniques allow affinity maturation of antibodies, and these techniques are being exploited to produce inhibitors for a number of toxins [6, 7]. The emphasis of this approach is on producing reagents with high affinity, based on the proposition that higher affinity will provide better protection.

However affinity, by itself, is a poor predictor of protective or therapeutic potential. Antibodies with high in vitro affinity for toxins do not automatically confer protection in vivo [8, 9] and may exacerbate the toxicity [10, 11]. The effects of using multiple antibodies with high affinities may be additive [12] or synergistic [8] or without effect [9]. In addition, epitope specificity [13], antibody titre [14–18], and dissociation rate [19] have been correlated with protection.

Toxins are produced by a number of plants, animals and microorganisms. Toxins may act at the cell surface and either damage the cytoplasmic membrane or bind to a receptor and act via transmembrane signalling subsequent to that binding [20]. Alternatively, toxins may cross the cell membrane and act on intracellular targets [20]. For example, anthrax lethal toxin, ricin and cholera toxin bind to a cell surface receptor and make use of cellular membrane trafficking to enter the cell [21, 22].

The objective of this study is to develop a simple mathematical model that may be used to predict the optimum antibody parameters (kinetic constants and concentration) needed to inhibit the binding of the toxin to its receptor. These predictions may be used to select candidate antibodies for progression to in vivo evaluation and to assess the potential value of affinity enhancement.

This paper is an extension to our previous work [23]. In the model presented in the following we explicitly take into account the process of toxin internalization and diffusive fluxes around the cell.

## 2. Model

The kinetic model describing the interactions of toxins with cell receptors can be formulated based on the well-known analytical framework for ligand-receptor binding. The models of this process have been studied for many years and a vast amount of literature has accumulated on this subject (see [24–28] and references therein).

When a toxin diffuses in the extracellular environment and binds to the cell surface receptors, the toxin concentration will vary in both space and time. Any rigorous description of this process would entail a system of Partial Differential Equations (PDE), which couples extracellular diffusion with reaction kinetics of the cell surface. The resulting system of PDE is nonlinear and too complex to be treated analytically. This complexity makes any comprehensive study of parameter optimization unfeasible. From another perspective, it is well known that under some rather broad conditions (see [24–28] and references therein) the reaction-diffusion system of the ligand-receptor binding can be well approximated by a system of Ordinary Differential Equations in which the spatial variability of the process is simulated by different concentrations of species in initially predefined spatial domains (called compartments). Although this compartment model is significantly simpler than the initial reaction-diffusion system, it still allows a consistent description of reaction-diffusion transport in underlying system [25, 26, 28]. In the current paper we use the compartment-model approach for our analytical study and numerical simulations.

**Figure 1:**
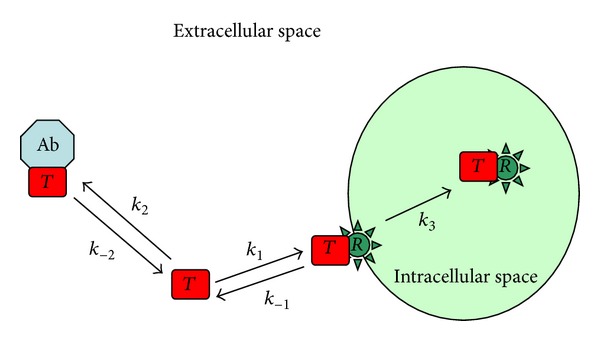
Schematic representation of the model for receptor-toxin-antibody interaction.

To begin, we consider the following simple model. The toxin, *T*, binds reversibly to cell surface receptors, *R*, with a forward rate *k*_1_ and a reverse rate *k*_−1_ to form the toxin-receptor complex *C*_*R*_ which is then slowly internalized at a rate *k*_3_. The neutralizing antibody binds competitively to the toxin with on and off rates of *k*_2_ and *k*_−2_, respectively. The antibody-toxin complex, *C*_*A*_, remains in the extracellular space (see Figure 1).

We can easily write an equation for the toxin-receptor binding (namely, without antibody being present). For a spherical cell of radius *a* with the toxin binding to its surface [24–28],

(1) $$\begin{array}{c} \frac{dC_{R}}{dt}=k_{f}^{e}RT+k_{r}^{e}C_{R}, \end{array}$$

where *C*_*R*_ is the concentration of the bound receptors (toxin-receptor complexes), *R* is the concentration of receptors, and *T* is the bulk toxin concentration (i.e., far from the cell surface) and is assumed to be spatially uniform. The effective forward and reverse rate coefficients are defined by [24–28]

(2) $$\begin{array}{c} k_{f}^{e}=\gamma k_{1},\;\;k_{r}^{e}=\gamma k_{-1}, \end{array}$$

where *k*_1_,  *k*_−1_ are intrinsic reaction rates, *k*_*D*_ = 4*πaD* is the diffusion rate, *D* is the diffusivity of toxin in the extracellular space, and *γ* = 1/(1 + *Rk*_1_/*k*_*D*_) ≤ 1 [25–27].

The bulk concentration of toxin *T* is mainly driven by the binding to antibody. Therefore, in this case we can write an equation system similar to (1) but without any “diffusive” modification of the intrinsic rate constants:

(3) $$\begin{array}{c} \frac{dC_{A}}{dt}=k_{2}AT+k_{-2}C_{A}, \end{array}$$

where *C*_*A*_ is the concentration of toxin-antibody complexes and *A* is the concentration of antibody.

The process of toxin internalization is phenomenologically introduced into our model by the following equation:

(4) $$\begin{array}{c} \frac{dT_{i}}{dt}=k_{3}C_{R}, \end{array}$$

where *T*_*i*_ is the concentration of internalized toxin. The corresponding term should be included in (1), so we arrive at modified expression for *k*_*r*_^*e*^

(5) $$\begin{array}{c} k_{r}^{e}=\gamma k_{-1}-k_{3}. \end{array}$$

The systems (1), (3), and (4) should be supplemented with three conservation laws for concentrations of *R*, *T*, and *A*:

(6) $$\begin{array}{c} R_{0}=R+C_{R}, \end{array}$$

(7) $$\begin{array}{c} A_{0}=A+C_{A}, \end{array}$$

(8) $$\begin{array}{c} T_{0}=T+C_{T}+C_{A}+T_{i}, \end{array}$$

where *R*_0_, *T*_0_, and *A*_0_ are the initial concentrations.

Equations (1), (3), (4), and (6)–(8) form a framework for our analysis. This is a system of nonlinear ODE (because of conservation laws (6)–(8) and because effective rates *k*_*f*_^*e*^, *k*_*r*_^*e*^ are functions of the receptor concentration). It can be easily solved numerically and also allows some analytical progress (see the following). If parameter *γ* ≪ 1 (and this is the case in many practical situations), then this model can be reduced to the “well-mixed” kinetic model with constant kinetic rates [23].

It is worth emphasizing that the aim of our analytical framework is to develop a simple but scientifically rigorous model that may be used to predict the optimum antibody kinetic properties and concentration required to achieve a desired protective effect rather than develop a detailed, biologically accurate model that captures all the details of the toxin internalization process. Therefore, the model does not take into account the pharmacokinetics of the toxin-antibody complex [11] or receptor internalization and recycling [29, 30]. *k*_3_ is a lumped constant that describes all the processes that result in the appearance of the free toxin in the intracellular space [31]. Wiley and Cunningham [32] and Shankaran et al. [33] have also developed mathematical models of this type of process.

We are particularly interested in the behaviour of the model under conditions most likely to reflect the real biological situation, that is, toxin concentration much lower than the concentration of receptors (*T*_0_/*R*_0_ ≪ 1).

Testing of the model was carried out using COPASI (software application for simulation and analysis of biochemical networks and their dynamics [34]) and the kinetic parameters for the binding of ricin to its receptor and its internalization [35] and competition by the monoclonal antibody 2B11 [8]. The kinetic parameters used are shown in Table 1. The value of *k*_3_ used is that determined by Sandvig et al. [35] to be the rate of irreversible binding of ricin to HeLa cells. For simplicity, the simulation was carried out using all reactions taking place in the same compartment.

To illustrate the model, we used toxin and receptor concentrations based on cell culture studies carried out in our laboratory. These typically use a cell concentration of 1 · 10^4^ cells per 100 *μ*L experiment and a ricin concentration of 10 pM. Assuming 3 · 10^7^ receptors/cell [35], the receptor concentration is approximately 5 nM.

## 3. Analytical Results

### 3.1. Cell Surface Binding

Initially we derive some analytical results for toxins that act at the cell surface and are not internalized; that is, we set *k*_3_ = 0 in (4). At equilibrium *d*/*dt* = 0 and from (1) and (3) we can write

(9) $$\begin{array}{c} C_{R}=\frac{RT}{K_{1}},\;\;C_{A}=\frac{AT}{K_{2}}, \end{array}$$

where *K*_1_ = *k*_1_/*k*_−1_, *K*_2_ = *k*_2_/*k*_−2_ are the association constants for the toxin binding to the receptor and antibody, respectively. It is worth noting that the parameter *γ* (diffusive correction of the intrinsic reaction rates) disappears from (9), so in this case the analytical results are identical to ones derived using the “well-mixed” approximation [23].

In order to simplify notations, we denote by *z* and *y* the equilibrium concentrations of the toxin-receptor and toxin-antibody complexes; that is,

(10) $$\begin{array}{c} z={\left[C_{R}\right]}_{\text{eq}},\;\;y={\left[C_{A}\right]}_{\text{eq}}. \end{array}$$

From (9) and conservation laws (6)–(8) the following closed equation for *z* can be derived:

(11) $$\begin{array}{c} \left(R_{0}-z\right)\left(T_{0}-z-y\right)-K_{1}z=0, \end{array}$$

(12) $$\begin{array}{c} y=A_{0}\frac{\epsilon z}{R_{0}-z\left(1-\epsilon \right)}, \end{array}$$

where *ϵ* = *K*_1_/*K*_2_.

Equation (11) can be written in a more conventional form of a cubic equation as follows:

(13) $$\begin{array}{c} a_{3}z^{3}+a_{2}z^{2}+a_{1}z+a_{0}=0, \end{array}$$

where

(14) $$\begin{array}{c} a_{3}=\epsilon -1,\\ a_{2}=\left(1-\epsilon \right)C_{0}+\epsilon A_{0}+R_{0},\\ a_{1}=-R_{0}\left(C_{0}+A_{0}+\left(1-\epsilon \right)T_{0}\right),\\ a_{0}=T_{0}R_{0}^{2}, \end{array}$$

and *C*_0_ = *R*_0_ + *K*_1_.

It is well known that (13) has a closed-form analytical solution (Cardano's formula [36]), which in our case provides a consistent way to derive exact solutions for the proposed model. Unfortunately these solutions still involve rather cumbersome expressions, which require further simplifications in order to be used in practical situations. In the following we present another approach that explicitly employs the smallness of ratio *T*_0_/*R*_0_ ≪ 1 and leads to a simple analytical expression for the protective properties of the antibody.

We observe that in the absence of antibody (i.e., *A*_0_ = 0), (11) is an elementary quadratic equation that has two roots. If we impose the obvious constraint *z* → 0 as *T*_0_ → 0, then there is only one solution, which we designate as *z*_0_:

(15) $$\begin{array}{c} z_{0}=\frac{C_{0}}{2}\left[1-{\left(1-\frac{4R_{0}T_{0}}{C_{0}^{2}}\right)}^{1/2}\right]. \end{array}$$

Under the condition *T*_0_/*R*_0_ ≪ 1, this can be simplified to

(16) $$\begin{array}{c} z_{0}\approx \frac{R_{0}T_{0}}{C_{0}},\;\;C_{0}=R_{0}+K_{1}. \end{array}$$

Let us now evaluate the effect of adding an antibody. From a mathematical point of view this effect (i.e., change of *z* under condition *A*_0_ > 0) is captured entirely by the term *y* in (11), so our aim is to provide a reasonable analytical estimation of this term.

From (12) and based on our initial assumption of low toxin concentration (*T*_0_/*R*_0_ ≪ 1), we can deduce the following simple estimate *y* ≈ *ϵzA*_0_/*R*_0_. This then leads to a modified form of (11) as follows:

(17) $$\begin{array}{c} \left(R_{0}-z\right)\left(T_{0}-z\right)-K_{*}z=0, \end{array}$$

where

(18) $$\begin{array}{c} K_{*}=K_{1}+\epsilon A_{0}. \end{array}$$

We can see that this is the same form as the equation for *z* when *A*_0_ = 0, but now with *K*_1_ replaced with *K*_∗_. This also implies that the analytical solution (16) is still valid but only with the substitution *K*_1_ = *K*_∗_.

In order to characterize the effect of an antibody on the binding of a toxin to its receptor, we introduce the nondimensional parameter Ψ, the relative reduction in *C*_*R*_ due to the introduction of an antibody as follows:

(19) $$\begin{array}{c} \Psi \equiv \frac{z\left(A_{0}>0\right)}{z\left(A_{0}=0\right)}. \end{array}$$

The analytical results presented previously enable us easily to derive a simple formula for the antibody efficiency parameter Ψ. By using (10), (16), (18), and (19), we can readily deduce the following:

(20) $$\begin{array}{c} \Psi =\frac{1}{1+\epsilon \lambda },\;\;\epsilon =\frac{K_{1}}{K_{2}},\;\;\lambda =\frac{A_{0}}{C_{0}}. \end{array}$$

This expression is the main result of the current paper and will be validated with numerical simulations.

To conclude this section let us briefly discuss some additional constraints for the parameters of our model in order for the expression (20) to be valid. As mentioned above the condition of low toxin concentration is always assumed in our study. Another simple condition can be derived from the constraint *C*_*R*_ + *C*_*A*_ ≤ *T*_0_ and by using (16):

(21) $$\begin{array}{c} \frac{R_{0}}{C_{0}}\left(1+\epsilon \frac{A_{0}}{C_{0}}\right)\approx \epsilon \frac{R_{0}A_{0}}{C_{0}^{2}}\leq 1, \end{array}$$

since *R*_0_/*C*_0_ ≤ 1. This condition could always be checked retrospectively and always hold in our numerical simulations.

### 3.2. Toxin Internalization

For toxins that are internalized, the effect of antibodies that prevent receptor binding is to reduce the effective rate of internalization. To examine and evaluate this effect, we need to analyze the full systems (1), (3), and (4).

In order to characterize the effect of antibody concentration on the rate of toxin internalization, we introduce a new parameter as follows:

(22) $$\begin{array}{c} G=\frac{T_{i}\left(A_{0}>0\right)}{T_{i}\left(A_{0}=0\right)}, \end{array}$$

which is a function of time (i.e., *G* ≡ *G*(*t*)).

Our aim is to deduce function *G* based on the kinetic models (1), (3), and (4). It is evident that *G* ≤ 1 for *t* > 0 and *G* → 1 as *t* → *∞* (since in that case all toxin will be internalized).

For the toxins of interest, while the receptor binding is rapid (time sale ~1/(*k*_1_ *C*_0_)) [24, 26], the subsequent internalization is much slower (time scale ~1/*k*_3_ ≫ 1/(*k*_1_ *C*_0_)). This coupling of slow and fast processes in our system allows us to develop a simplified model of toxin internalization using the well-known framework of Quasi-Steady-State Approximation (QSSA); see [24–28, 37] and refs therein.

When applied to our system, QSSA elucidates the toxin internalization as a two-stage process. After the initial rapid binding of the toxin to the receptor we can simply set *dC*_*R*_/*dt* = 0 in (1). The further slow evolution of *T*(*t*) (namely, quasi-steady state) is completely determined by the conservation laws (8) and (4) and spans a time scale of the order of the internalization time (~1/*k*_3_). In addition, for solving (4) at the initial stage of internalization, we can assume that *T*_*i*_ ≪ *T*_0_ and write

(23) $$\begin{array}{c} T_{i}\left(t\right)=k_{3}z_{0}t,\;t\ll \frac{1}{k_{3}}, \end{array}$$

where *z*_0_ is given by expressions (15) and (16). The evolution of *T*_*i*_(*t*) for the late stage of internalization can be readily derived from (4) and (6)–(8) by assuming [*T*_0_ − *T*_*i*_(*t*)] ≪ *T*_0_:

(24) $$\begin{array}{c} T_{i}\left(t\right)=T_{0}\left[1-\exp\left(-k_{3}t\right)\right],\;t\geq \frac{1}{k_{3}}, \end{array}$$

so *T*_*i*_(*t*) exponentially approaches its saturation limit. A simulation of this process is shown in Figure 5, and the slow linear increase of *T*_*i*_ at the initial stage is clearly visible.

Now, consider the case where *A*_0_ > 0. According to (23) the main effect of the introduction of an antibody is to reduce the value of *z*_0_, as described in the previous section. Then, based on (22), (23), and (19) we can conclude that, during the quasi-equilibrium stage, the following approximation holds:

(25) $$\begin{array}{c} G=\frac{T_{i}\left(A_{0}>0\right)}{T_{i}\left(A_{0}=0\right)}\approx \Psi , \end{array}$$

where Ψ is given by expression (20).

The overall effect of introducing an antibody can be best described in terms of the internalization half-time, *τ*_*i*_. Without antibody the latter can be estimated from (24) and condition *T*_*i*_(*τ*_*i*_) = *T*_0_/2. Thus from (23) we yield

(26) $$\begin{array}{c} \tau_{i}\approx \frac{T_{0}}{2k_{3}z_{0}}=\frac{C_{0}}{2k_{3}R_{0}}. \end{array}$$

For the internalization time with the presence of antibody we can apply reduced value of *z*_0_ and write the following simple formula:

(27) $$\begin{array}{c} \frac{\tau_{i}}{\tau_{i}^{0}}\approx \frac{1}{\Psi }, \end{array}$$

where *τ*_*i*_^0^ is the internalization time in the absence of antibody (*A*_0_ = 0).

Equations (26) and (27) have a clear interpretation. As described in the previous section, the introduction of an antibody results in a decrease, at *t* ≪ *τ*_*i*_, in the equilibrium value of *C*_*R*_ (i.e., in *z*_0_). This can be related, in accordance with (23) and (26), to a corresponding decrease in the concentration of internalized toxin *T*_*i*_ and a consequent increase in the toxin internalization time (since it takes longer to achieve a give level of *T*_*i*_). Since changes in *z*_0_ can be described comprehensively by means of the parameter Ψ, it still remains the only parameter needed to characterize the influence of an antibody on the concentration of internalized toxin (25), (27).

It is evident that the two main effects described above (reduction of the concentration of internalized toxin at a given time and increase in the time required for the internalized toxin to reach a given concentration) are not independent of each other. The linear relationships (25), (27) allow us to establish a general identity that relates these two effects for any time *t*.

Let us assume that for *A*_0_ = 0, *τ*_0_ is the time taken for the internalized toxin to reach a concentration *T*_*i*_^0^ (i.e., *τ* ^0^ = *T*_*i*_^0^/(*k*_3_ *z*_0_); see (23)). The effect of introducing an antibody is to reduce the internalized toxin concentration to a value *T*_*i*_ ≤ *T*_*i*_^0^. Then from (25), (27) we can derive the following identity:

(28) $$\begin{array}{c} T_{i}\tau_{i}=T_{i}^{0}\tau_{i}^{0}, \end{array}$$

where *τ*_*i*_ is the time required for the internalized toxin to reach *T*_*i*_^0^ when *A*_0_ > 0. The identity (28) has no explicit dependency on antibody kinetic parameters or concentration and provides an easy way to calculate any of the parameters (*T*_*i*_, *T*_*i*_^0^, *τ*_*i*_, *τ*_*i*_^0^) if the other three are known.

## 4. Numerical Results and Discussion

We have derived an analytical expression for the parameter Ψ, the relative ability of an antibody to reduce the binding of a toxin to its receptor (20). Our derivation is based on the following assumptions:

1. toxin concentration is much lower than the receptor concentration,
2. for toxins that are internalized, the internalization rate is much slower than establishment of the receptor-toxin binding equilibrium.

Applying these assumptions, we found that parameter Ψ is independent of the toxin concentration (see (20)); that is, it is determined by the ratio of antibody to receptor concentration and not by the ratio of antibody to toxin concentration as commonly used. For the low toxin/receptor ratios likely to occur in biological situations, the condition (21) can be met by large range of antibody kinetic parameters. From this point of view (20) should be valid for most practical applications.

**Figure 2:**
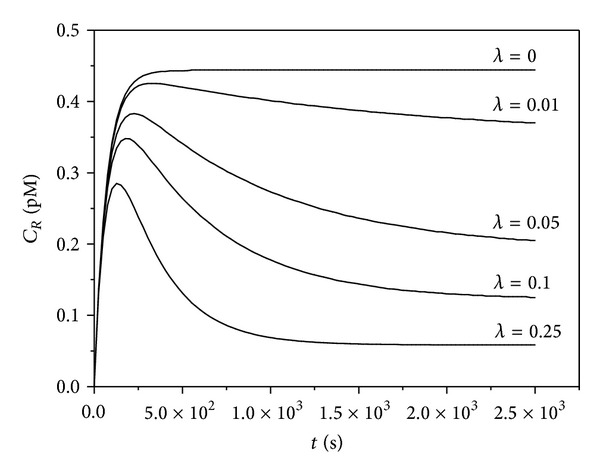
Simulated effect of antibody concentration on formation of toxin-receptor complexes *C*_*R*_. Parameter *λ* = *A*_0_/*C*_0_, *C*_0_ = *R*_0_ + *K*_1_. The binding curves were created using the simulation package COPASI and the kinetic constants in Table 1. *R*_0_ = 5 nM,  *T*_0_ = 10 pM, *C*_0_ = 1.15 · 10^−7^.

 The implications of our analytical findings are illustrated by simulation of the complete kinetic models ((1),(3), (4), and (6)–(8)) using the kinetic constants for ricin and the anti-ricin antibody 2B11 (Table 1).  Figure 2 is a simulation of the effect of the presence of an antibody on the binding of the toxin to its receptor (formation of *C*_*R*_). The antibody concentration is expressed as the dimensionless parameter *λ* = *A*_0_/*C*_0_. In this case, since *R*_0_ and *T*_0_ ≪ *K*_1_, the parameter *C*_0_ = *R*_0_ + *K*_1_ is dominated by *K*_1_ (1.08 · 10^−7^).

**Figure 3:**
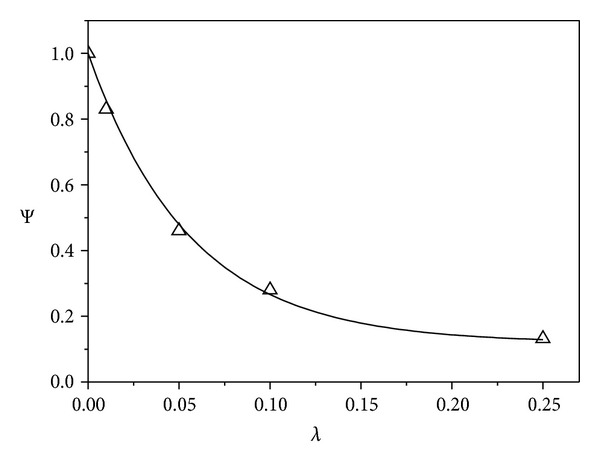
Effect of antibody concentration on protection factor. Parameter Ψ (19) was determined from (20) (solid lines) and by using simulated values of *C*_*R*_ from Figure 2 at 2500 sec (△),  *ϵ* = 25.9.

Figure 3 shows the effect of increasing antibody concentration on Ψ. There is a good agreement between the values of Ψ determined from (20) and from (19) using the equilibrium values of *C*_*R*_ determined from simulation of the complete kinetic model (Figure 3). For instance, the results predict that, for this toxin and antibody combination, the additional protection provided by increasing the antibody concentration diminishes rapidly when *λ* exceeds 0.1.

**Figure 4:**
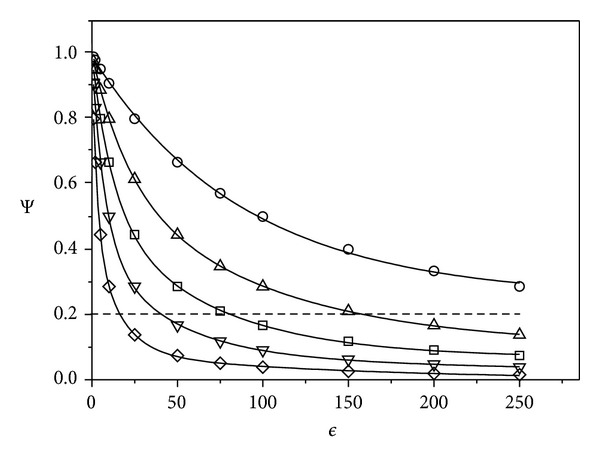
Protection factor Ψ (19) as a function of parameter *ϵ* = *K*_1_/*K*_2_ and *λ* = *A*_0_/*C*_0_ ((20)): *λ* = 0.01 (*⚪*); 0.025 (△); 0.05 (□); 0.1 (*▽*); 0.25 (*◊*). The range of values for *λ* and *ϵ* below dashed line corresponds to 80% protection.

Figure 4 shows the relationship (20) between Ψ, antibody concentration and the toxin/antibody and the ratio of toxin/receptor dissociation constants (*ϵ*). This plot is valid for all combinations of toxin, receptor, and antibody consistent with the assumptions used to derive (20), principally *T*_0_ ≪ *R*_0_. The antibody kinetic parameters and concentration required to provide a specified degree of protection may be determined from this plot. For example, any combination of *ϵ* and *λ* falling below the dashed line will reduce either *C*_*R*_ or *T*_*i*_ by 80%. 

 This, in turn, enables important judgements to be made about antibody selection. For example, if an antibody concentration of 0.25*C*_0_ (*λ* = 0.25) is achievable, then an antibody with an *ϵ* value of 50 will provide good protection (93% reduction in *C*_*R*_ or *T*_*i*_). If an antibody concentration of only 0.05*C*_0_ (*λ* = 0.05) is achievable, then an *ϵ* value of 250 is required to achieve the same level of protection. The structure of (20) is such that a given increase in protection (Ψ or Γ) may be achieved by either an *x*-fold increase in *ϵ* or an *x*-fold increase in *λ*.

**Figure 5:**
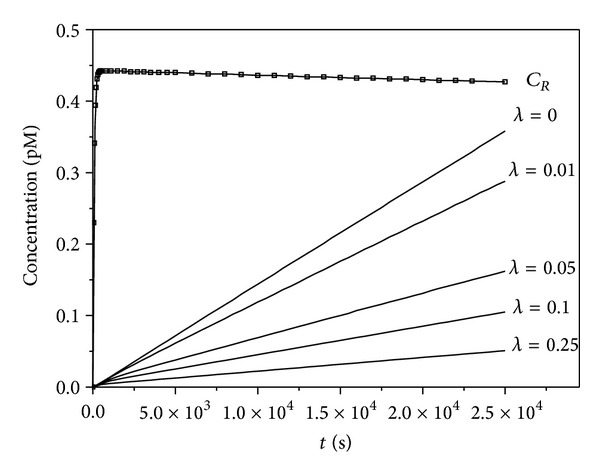
Different time scales for formation of receptor-toxin complex *C*_*R*_ (□) and associated toxin internalization *T*_*i*_ (solid lines). Results of COPASI simulation with kinetic constants from Table 1. *λ* = *A*_0_/*C*_0_, *R*_0_ = 5 nM, *T*_0_ = 10 pM, *C*_0_ = 1.15 · 10^−7^, *ϵ* = 25.9.

**Figure 6:**
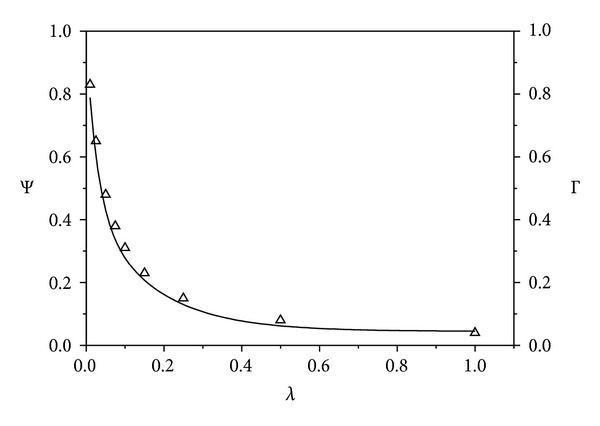
Comparison of parameters Ψ and Γ. Γ (△) was determined using values of *T*_*i*_ and *T*_*i*_^0^ at *t* = 10^4^ sec from toxin internalization time courses simulated using COPASI and the kinetic constants in Table 1. Parameter Ψ (solid line) was determined from (20). *R*_0_ = 5 nM, *T*_0_ = 10 pM, *C*_0_ = 1.15 · 10^−7^, *ϵ* = 25.9.

The effect of antibody on toxin internalization is simulated in Figure 5. Rapid equilibration of receptor and toxin is followed by slow accumulation of toxin within the cell. Equation (25) predicts that Ψ is the only parameter needed to characterize the influence of an antibody on toxin internalization.  Figure 6 compares Γ calculated using (25), (20) with Γ determined using values of *T*_*i*_ and *T*_*i*_^0^ at *t* = 10^4^ sec from this simulated data and shows good agreement between the two values under the condition *T*_0_ ≪ *R*_0_, although the value of Γ is slightly greater than Ψ. The plot predicts the degree of protection provided by a given concentration of antibody and enables assessment of the value of increasing antibody concentration beyond a certain value. For example, to enhance the reduction of *T*_*i*_ from 90% to 95% requires doubling of *A*_0_.

**Figure 7:**
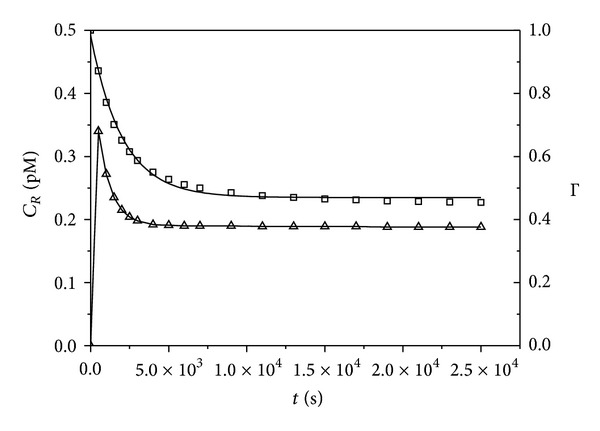
Establishment of the quasi-equilibrium state in the presence of antibody. *C*_*R*_ formation (△) was simulated using COPASI and the kinetic constants in Table 1. Γ (□) was determined using (25) and values *T*_*i*_ and *T*_*i*_^0^ at *t* = 10^4^ sec using simulated toxin internalization time courses. *R*_0_ = 5 nM, *T*_0_ = 10 pM, *C*_0_ = 1.15 · 10^−7^, *λ* = 0.05.

The expression for Ψ, (20), assumes a quasi-equilibrium state in the system. In practice, this state may take significant time to be achieved.  Figure 7 shows a simulation of the time taken by the ricin/receptor/2B11 system to reach the quasi-equilibrium state for *λ* = 0.05. The value of Γ determined from the toxin internalization profiles (Figure 7) parallels this process; that is, experimental validation of Γ must allow sufficient time to elapse for the quasi-equilibrium state to be established.

**Figure 8:**
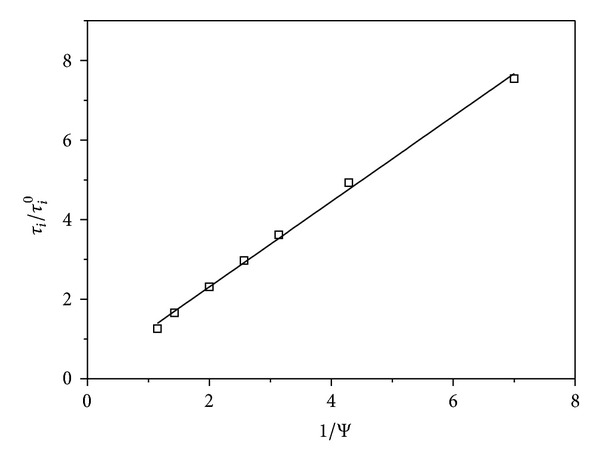
Relationship between toxin internalization time *τ*_*i*_ and protection factor Ψ (19). Solid line is formula (27) and (□) is simulation with COPASI. *τ*_*i*_ was determined as the time to internalize 5 · 10^−14^ M of ricin. All other parameters are the same as in Figure 7.

The relationship between the internalization time *τ*_*i*_ and Ψ described in (27) is shown in Figure 8. Ψ was determined from simulated toxin internalization time courses (Figure 5) as the time to internalize 5 · 10^−14^ M ricin. The slope of the fitted line is 1.07, close to the predicted value of 1.0.

In summary, the protection provided by an antibody against toxins that act either at the cell surface or after binding to the cell surface followed by internalization may be predicted from a simple kinetic model. Protection parameter Ψ is a simple function of antibody, receptor, and toxin concentrations and the kinetic parameters governing the binding of the toxin to the receptor and antibody:

(29) $$\begin{array}{c} \Psi =\frac{1}{1+\left(K_{1}/K_{2}\right)\left(A_{0}/C_{0}\right)}. \end{array}$$

The calculated value of Ψ matches closely the degree of protection determined from numerical simulation of the binding and internalization reactions and provides a convenient method for predicting the optimum antibody parameters (concentration and dissociation constants) needed to provide effective treatment or prophylaxis for toxins.

## Figures and Tables

**Table 1:** Kinetic constants used in numerical simulations (the binding of ricin to its receptor and the monoclonal antibody 2B11).

Reaction	Value
*k*_1_	1.3 · 10^5^ M^−1^s^−1^
*k*_−1_	1.4 · 10^−2^ s^−1^
*k*_2_	1.25 · 10^5^ M^−1^s^−1^
*k*_−2_	5.2 · 10^−4^ s^−1^
*k*_3_	3.3 · 10^−5^ s^−1^
